# Cæsarian Section

**Published:** 1856-10

**Authors:** 


*Cæsarian Section.*—Among the notable events of the last few months, we should not neglect to include the first Cæsarian operation ever performed in this State, resulting in the recovery of both mother and child. This surgical feat has been achieved by our friend and fellow-townsman, Dr. Charles S. Mills, who performed the operation of hysterotomy on the person of a negro dwarf, during the month of May last, with entire success.

The subject of this operation is about twenty-three years old, and not more than three feet nine inches in height. The upper portion of the body is very well developed, the arrest of growth being confined entirely to the pelvis and lower limbs. The probable necestity for the operation had led the family physician to propose the induction of premature delivery; but his advice was not followed, and on arriving at full term, the extreme contraction of the pelvis prevented any doubt as to the true course to be pursued.

The operation, which was performed under the influence of chloroform, and at night, presents no features of peculiar interest. The happy effects of chloroform were exemplified in a marked degree, the viscera remaining perfectly quiet, not interfering as they usually do, in the progress of the operation. The case went on safely, without any symptoms likely to produce alarm, until crural phlebitis appeared about the eighteenth day after the operation, which, however, yielded to active treatment, and the woman, by the

7th of June, had entirely convalesced. During this period the infant had never required the interference of the physician.

It is with much pleasure that we record the entire success of this operation, the first to be found in the annals of Virginia surgery; and we will close these remarks with a short sketch of the various Cæsarian sections performed in this State, which we have been able at this time to collect, and their results.

Dr. Herndon, of Fredericksburg, in the year 1846, performed this operation for the purpose of taking away a dead fœtus from the womb. This case is remarkable for the fact, that on making the abdominal section, the peritonæum was found closely adherent to the uterus, and hence the operation was rendered remarkably simple, as the integrity of the abdominal cavity proper was not disturbed. The dead fœtus was extracted, and the woman *recovered.* This case is reported in the April No., 1846, of the American Journal of Medical Sciences.

Dr. Smith, of Northampton county, Virginia, performed the operation of hysterotomy, in or near the town of Eastville, sometime during the year 1851. The particulars of this case will be reported in a future number of this journal. It resulted in the death of the mother, but the *child lived.*

The case of Dr. Mills, is the next in order, securing the safety of both mother and child; and a few weeks subsequent to the date of this operation, Dr. Edward Drew, of this city, was compelled to perform the Cæsarian section on the body of a negro dwarf whose pelvis was so extremely contracted as to prevent the possibility of a natural delivery. This case also will appear in our columns. It resulted in the death of the mother, the life of the child being saved.

Thus, in four operations, we have the recovery of the mother in two instances, and three children saved—the fourth (Dr. Herndon's case, having been dead for some time. We think it probable, however, that in a few months we will be able to collect the statistics of other operations of like character, which have been performed in Virginia in times gone by, and have, with characteristic neglect, been permitted to remain unrecorded.

The success of this operation in the hands of American surgeons, has been remarkably great. Churchill, in his Tables of the Results of British and American Practice, gives *five* American operations, including Dr. William Gibson's famous case, on whom hysterotomy was performed *twice successfully.* These five cases resulted in the recovery of every mother and the safety of four children. But in forty-seven British operations, we observe a loss of thirty-eight mothers!

The statistics of the European operations are more encouraging. In all Catholic countries, we find a greater inclination to the performance of this operation at an early period, which may be attributed to the desire to preserve the life of the unbaptized infant. Indeed, there are cases of women who have submitted to the Cæsarian section, and afterward have borne children per vias naturales. In the dictionarie of Fabre, we find two hundred and ninety-three cases collected by Velpeau and Hœbeke. Of these, one hundred and thirty-four were saved. Thus, on the continent, two-fifths of the women have recovered, and about three-fourths of the children saved.—*Virginia Med. Jour.*

